# ‘Clean’ hydrolase reactions using commercial washing powder†

**DOI:** 10.1039/c9ra05828a

**Published:** 2019-08-02

**Authors:** Jie Zhang, Fabio Tonin, Wuyuan Zhang, Peter-Leon Hagedoorn, Lloyd Mallée, Frank Hollmann

**Affiliations:** Chongqing Key Laboratory of Catalysis & Functional Organic Molecules, College of Environment and Resources, Chongqing Technology and Business University Chongqing 400067 China; Department of Biotechnology, Delft University of Technology Van der Maasweg 9 2629HZ Delft The Netherlands F.Hollmann@tudelft.nl

## Abstract

We report the use of commercial laundry powder as a biocatalyst for a range of lipase-catalysed reactions including (trans)esterification, ester hydrolysis and chemoenzymatic epoxidation reactions. The enzymatic laundry powder exhibited excellent stability and recyclability, making it a readily available and cheap biocatalyst for chemical transformations.

## Abstract 2

We report the use of commercial laundry powder as a biocatalyst for a range of lipase-catalysed reactions including (trans)esterification, ester hydrolysis and chemoenzymatic epoxidation reactions.

## Graphical abstract

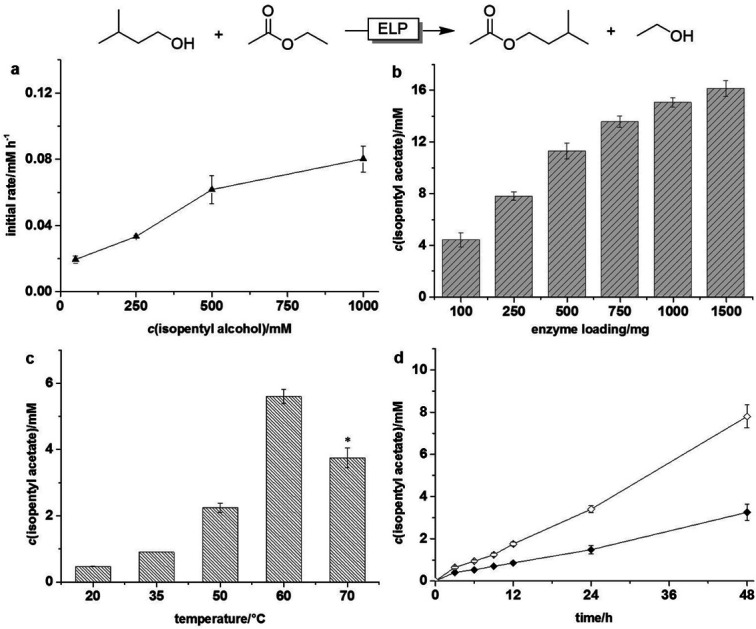

Modern laundry powders are complex compositions consisting of much more than just surfactants containing more than 25 different ingredients.^1^ Amongst them, enzymes are frequently included. Enzymes increase the washing performance by degrading poorly water soluble polymeric carbohydrates, proteins and triglycerides. Particularly hydrolytic enzymes such as cellulases, amylases, proteases and lipases are found in so-called enzymatic laundry powders (ELPs).^2^ Having this in mind, ELP may also be considered as a cheap and readily available source of biocatalysts. Hence, they may be considered as affordable catalysts *e.g.* for education purposes or for research groups with limited financial possibilities. In fact, ELPs have been used to isolate DNA from tissue,^3^ gels,^4^ blood,^5^ or hair.^6^

Preparative applications of ELPs, however, have so far not been considered. In this respect, especially the lipases contained in ELPs represent an interesting starting point. Today, lipases are established catalysts for the synthesis of a broad range of compounds ranging from chiral alcohols and amines through kinetic resolution of racemic starting materials^7^ to cosmetic esters (Scheme 1).^8^ Also so-called perhydrolysis reactions yielding peracids to mediate chemical oxidation reactions are gaining increasing attention (Scheme 1).^9,10^ We therefore set out to explore the use of ELP to catalyse some typical lipase reactions.

**Scheme 1:**
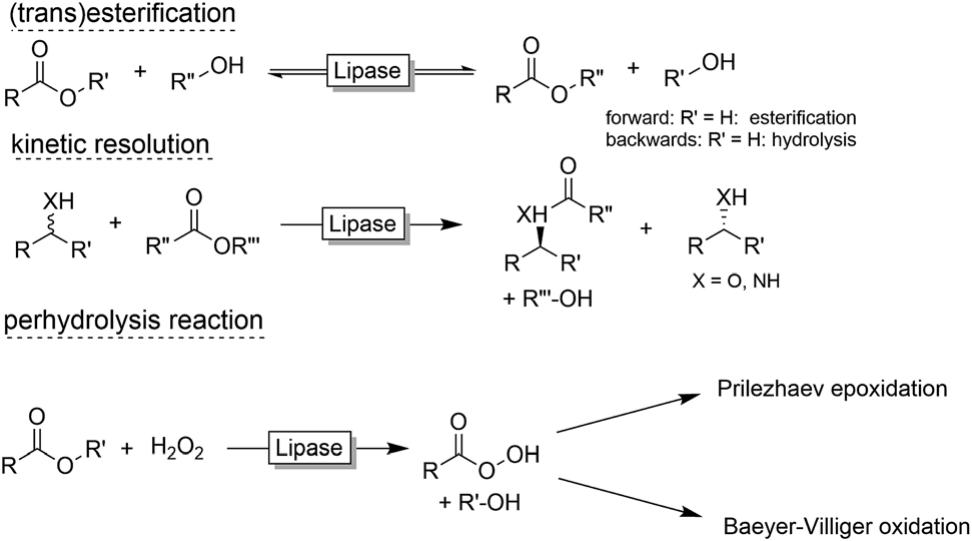
Selection of typical, preparative applications of lipases in organic synthesis.

For biocatalyst preparation we used a commercially available heavy-duty detergent powder purchased from a local supermarket and used it without any further treatment.

As a first model reaction we chose the transesterification between ethyl acetate with isopentyl alcohol (Fig. 1). For this, the ELP was suspended in a mixture of both reagents in the absence of any further solvent. It is worth mentioning here that performing this (and all subsequent reactions) in the absence of ELP or using thermally treated ELP (see ESI† for details on the thermal enzyme inactivation procedure) gave trace amounts of product only. Therefore, we can assume that all reactions reported here are indeed enzymatic reactions.

**Fig. 1:**
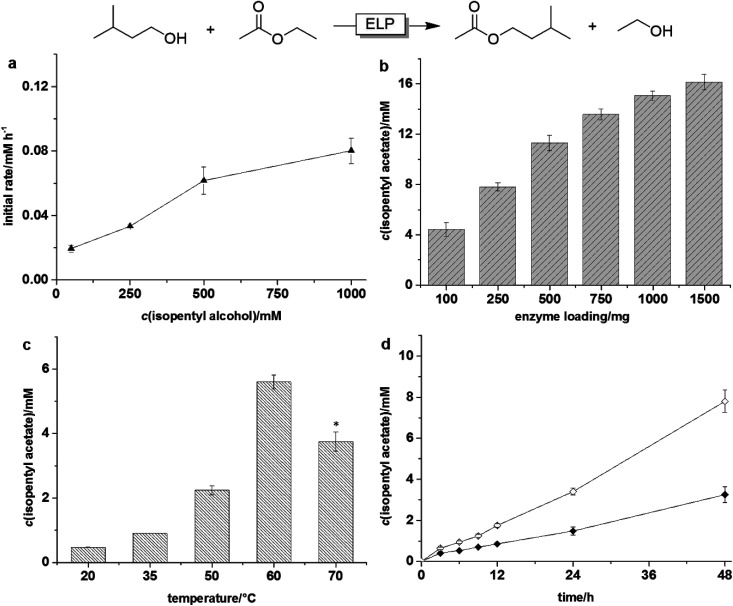
ELP driven transesterification of ethyl acetate to isopentyl acetate. (a) The rate dependency of the isopentyl acetate production on isopentyl alcohol concentration. Conditions: 1000 mg ELP, 60 °C, shaking speed 180 rpm, 24 h. (b) Effect of ELP loading on the transesterification of ethyl acetate. Conditions: *c*(isopentyl alcohol) = 500 mM, 60 °C, rotating speed 99 rpm, 72 h. (c) Effect of temperature on the isopentyl acetate formation. Conditions: *c*(isopentyl alcohol) = 500 mM, 1000 mg ELP, rotating speed 99 rpm, 36 h. (d) Time course of the isopentyl acetate formation performed by shaking (◆) and rotating (◇). Conditions: *c*(isopentyl alcohol) = 500 mM, 1000 mg ELP, 60 °C, shaking speed 180 rpm or rotating speed 99 rpm. All reactions using ethyl acetate as solvent (*ca.* 20 eq. on isopentyl alcohol) were performed in a reaction scale of 10 mL. *: Reactions were performed in an oil bath with stirring speed 300 rpm. Error bars indicate the standard deviation of duplicate experiments (*n* = 2).

A preliminary characterisation of the parameters influencing the ELP-catalysed transesterification reaction revealed that the system is very well behaved. Increasing the concentration of the acyl donor positively influenced the product formation rate (Fig. 1a). The same is true for the catalyst loading (Fig. 1b). The reaction rate steadily increased with increasing ELP concentrations. Also the influence of the reaction temperature on the productivity of the transesterification reaction was in line with our expectations exhibiting an optimal value at 60 °C, which is also the laundry temperature recommended by the producer.

Quite interestingly, the method of physical mixing the reaction mixture had a significant effect on the reaction rate and changing from horizontal to orbital shaking (Fig. S1 and S3†) enhanced the reaction rate more than two-fold (Fig. 1d). We therefore assumed that diffusion-limitation may be overall rate-limiting. Interestingly enough, decreasing the particle size of the ELP by mechanical grinding, had no significant effect on the reaction rate (Fig. S5†).

The preparative applicability of ELP was exemplarily demonstrated in the synthesis of isopentyl acetate on litre-scale (Fig. 2). Over at least 12 days, the product accumulated linearly indicating a very high stability of the enzyme under the comparably harsh reaction conditions. Overall, 1.02 g (isolated product) were obtained from this reaction (Fig. S7 and S8†). Admittedly, the catalyst performance in this reaction was not most convincing (150 g ELP per gram of product within 12 days). We therefore investigated the recyclability of the ELP under reaction conditions. Very pleasingly, the ELP showed excellent recyclability. As shown in Fig. 3, the ELP could be recycled at least 8 times maintaining 89.7 ± 1.1% of its initial transesterification activity.

**Fig. 2:**
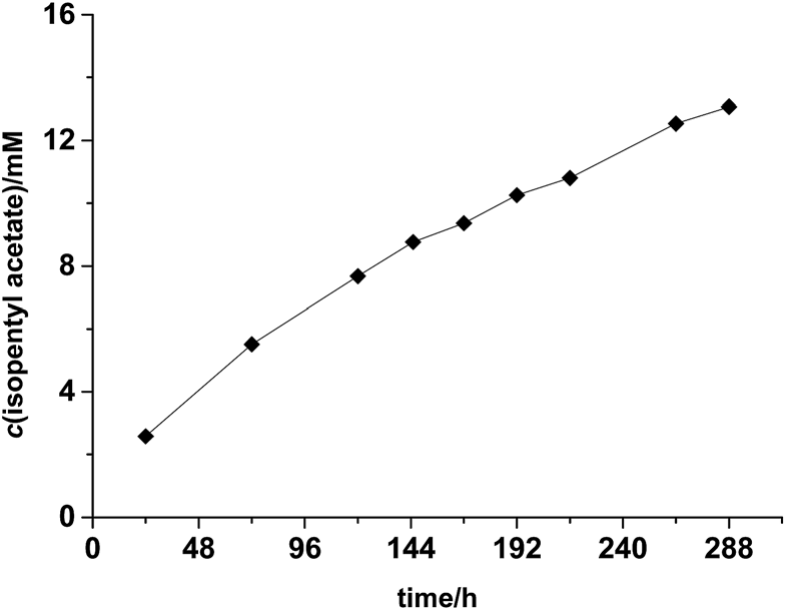
Time course of the preparative-scale synthesis of isopentyl acetate formation using ELP. Conditions: 1 L reaction scale, *c*(isopentyl alcohol) = 500 mM, ethyl acetate as solvent (*ca.* 20 eq.), 150 g ELP, 60 °C, stirring speed 400 rpm. Error bars indicate the standard deviation of duplicate experiments (*n* = 2).

**Fig. 3:**
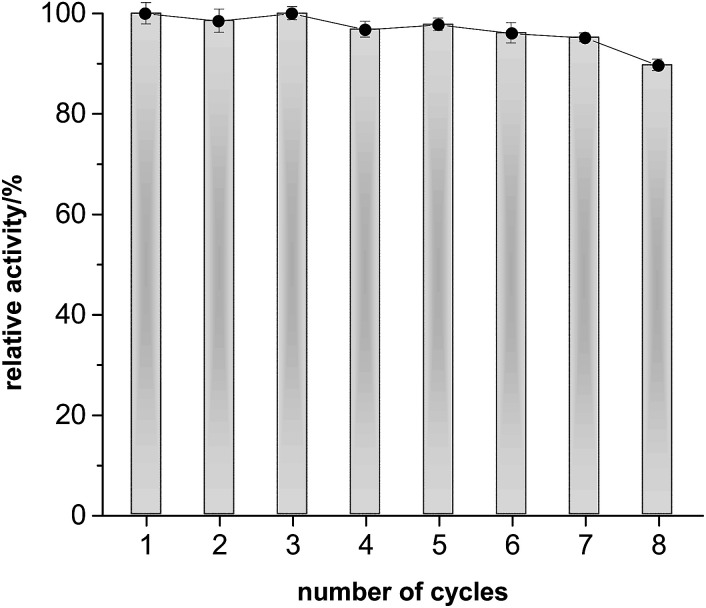
Recyclability of ELP for transesterification of ethyl acetate to isopentyl acetate. Relative activity = *c*(isopentyl acetate, cycle run) × *c*(isopentyl acetate, first run)^−1^. Conditions: 10 mL reaction scale, *c*(isopentyl alcohol) = 500 mM, ethyl acetate as solvent (*ca.* 20 eq.), 1000 mg ELP, 60 °C, rotating speed 99 rpm, 48 h for one cycle. Error bars indicate the standard deviation of duplicate experiments (*n* = 2).

Encouraged by these results, we further explored the substrate scope of the ELP-catalysed transesterification and hydrolysis reactions (Table 1). Depending on the steric demand of the starting material, good to poor reaction yields were obtained suggesting that ELP may be practical catalysts for at least a range of different esters.

**Table 1:** Substrate scope of ELP driven transesterification, esterification and hydrolysis reactions^a^

Entry	Substrate	Product	*c*(product)/mM
1^b^	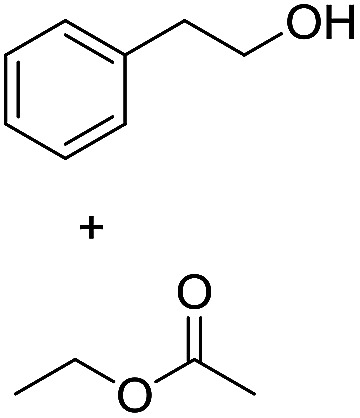	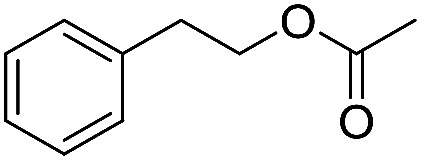	7.8 ± 0.6
2^b^	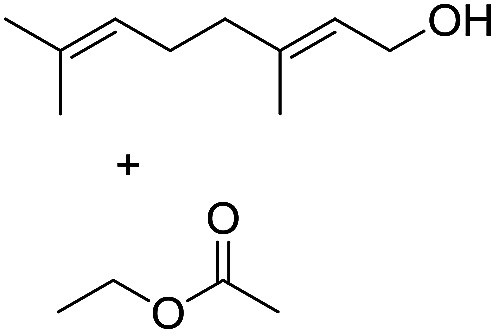	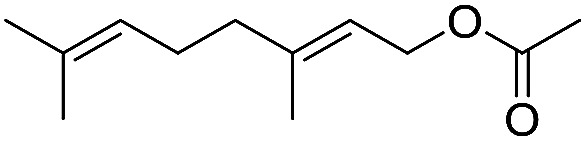	9.2 ± 0.2
3^c^	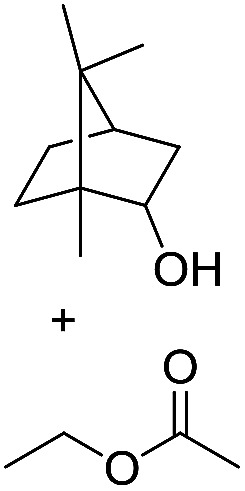	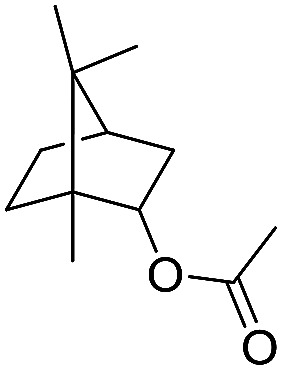	0.3
4^b^	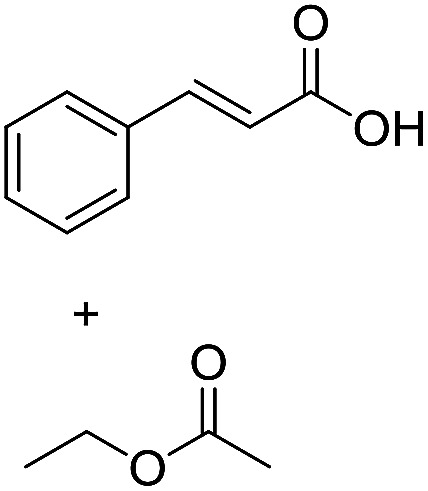	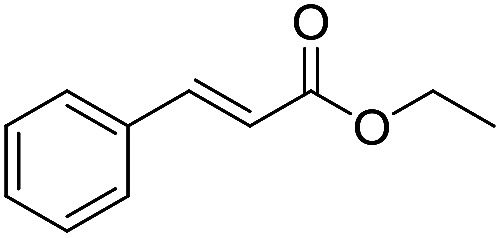	4.6 ± 0.4
5^b^	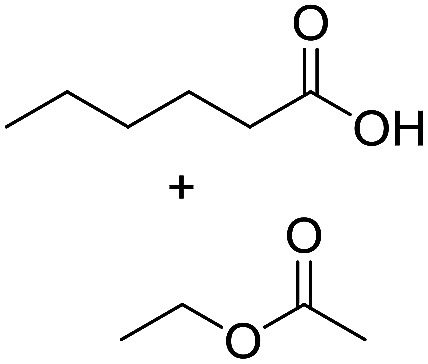	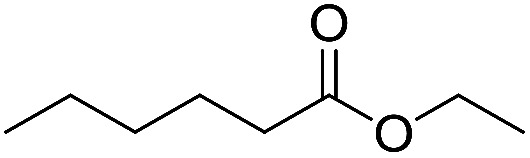	0.7
6^b^	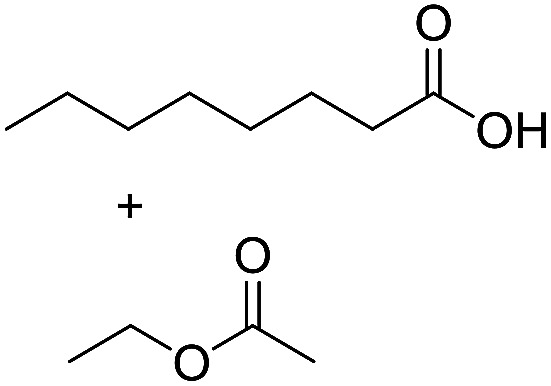	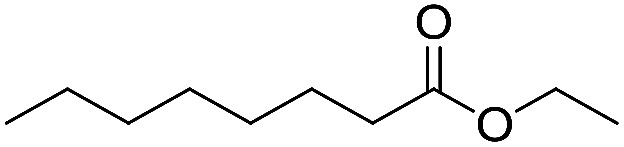	0.7
7^d^	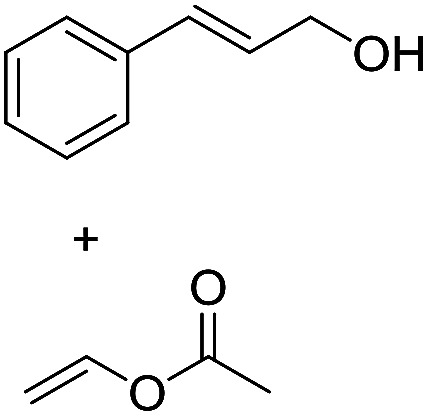	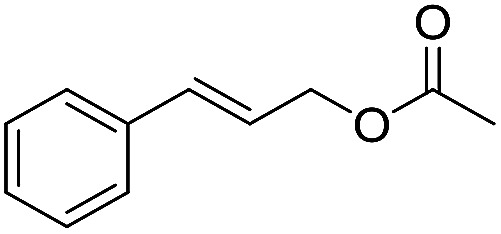	6.0
8^e^	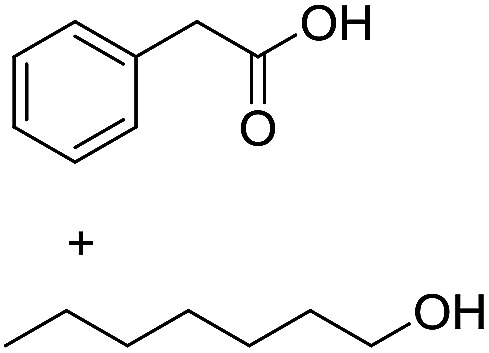	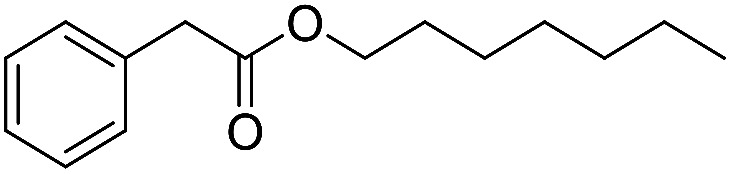	2.0
9^f^	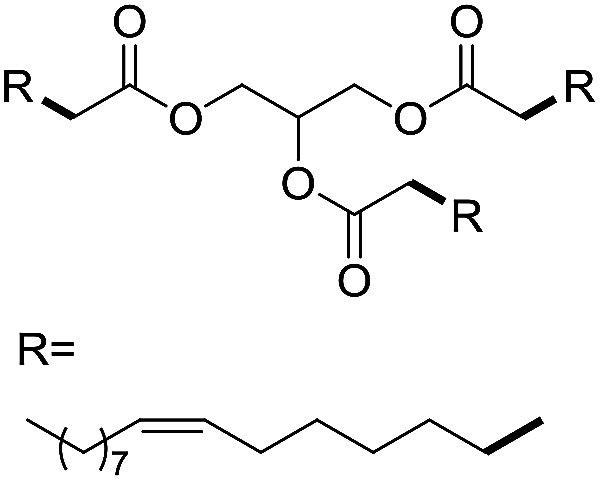	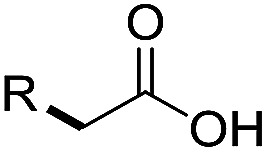	6.2 ± 0.3

a Reaction conditions: 10 mL reaction scale, 1000 mg ELP, 60 °C, rotating speed 99 rpm, 48 h.

b *c*(substrate) = 500 mM, ethyl acetate as solvent.

c *c*(substrate) = 100 mM, ethyl acetate as solvent.

d *c*(cinnamyl alcohol) = 10 mM, *c*(vinyl acetate) = 20 mM, toluene as solvent, stirring speed 500 rpm.

e *c*(phenylacetic acid) = 10 mM, *c*(1-heptanol) = 20 mM, toluene as hydrophobic solvent for phenylacetic acid, stirring speed 500 rpm.

f 2 mL triolein + 20 μL H_2_O, 200 mg ELP, stirring speed 500 rpm. Error bars indicate the standard deviation of duplicate experiments (*n* = 2).

We also tried the ELP as catalyst for the kinetic resolution of racemic 1-phenyl ethanol. Though only one product enantiomer was observed in the reaction (Fig. S9†), indicating an exclusive enantioselectivity, the very low productivity of the reaction (*i.e.* the conversion of the secondary alcohol) was discouraging.

Finally, we investigated the applicability of the ELP as catalyst for the epoxidation of styrene. Ever since the first report by Björkling *et al.*,^9^ chemoenzymatic epoxidation reactions using lipases is enjoying an even increasing popularity.^10^ Since modern heavy-duty detergents also contain significant amounts of inorganic peroxides we also tested the application of the chemoenzymatic Prilezhaev reaction on styrene (Table 2).

**Table 2:** Epoxidation of styrene by ELP^a^

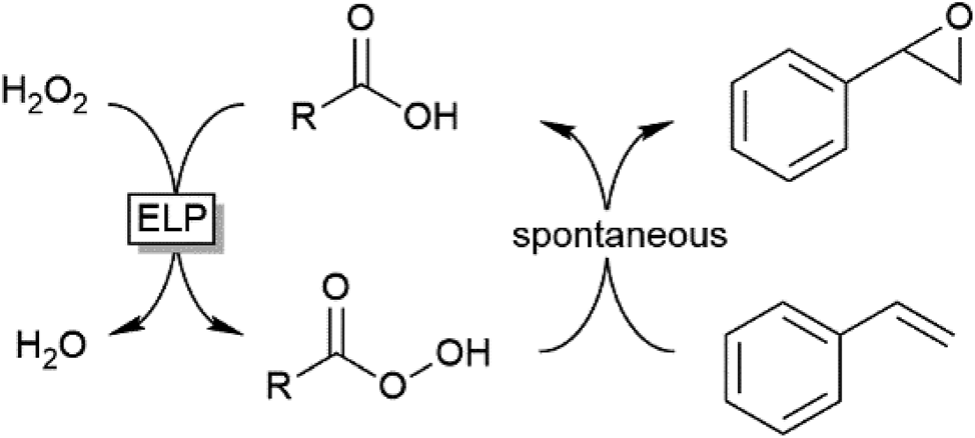	
Reactions	*c*(styrene oxide)/mM
Styrene only	3.6
Styrene + octanoic acid^b^	6.2

a Reaction conditions: 10 mL styrene solution, *c*(styrene) = 500 mM, toluene as solvent, 1000 mg ELP, 60 °C, stirring speed 500 rpm, 48 h.

b *c*(octanoic acid) = 100 mM.

The chemoenzymatic Prilezhaev reaction necessitates, next to the lipase catalyst, hydrogen peroxide and a carboxylic acid co-catalysts (being transformed into the corresponding peracid to perform the epoxidation reaction). As shown in Table 2, ELP apparently contains all these components as very significant production of styrene oxide was observed in the presence of the ELP alone. Adding more carboxylic acids improved the epoxide yield. It is interesting to note that the ELP-contained peroxide was available for the lipase-catalysed formation of the peracid even under non-aqueous conditions.

## Conclusions

With this contribution we aimed at demonstrating the synthetic applicability of commercially available heavy duty detergent compositions for the synthesis of esters and epoxides representing some typical lipase reactions. Modern ELPs contain all components needed for these reactions and therefore constitute a very cost-efficient alternative to expensive enzyme preparations. We are convinced that further, classical hydrolase reactions are possible using ELPs.

This contribution may inspire school or university teachers and may be an alternative for research groups with limited financial possibilities to utilise readily available and cheap laundry compositions.

## Conflicts of interest

There are no conflicts to declare.

## Supplementary Material

RA-009-C9RA05828A-s001
