# Supplementary material for: ‘Clean’ hydrolase reactions using commercial washing powder
Source: RSC Adv. 2019 Aug 2;9(42):24039–42. doi: 10.1039/c9ra05828a (PMC9069458; doi:10.1039/c9ra05828a)
Supplement: RA-009-C9RA05828A-s001 [file RA-009-C9RA05828A-s001.pdf]

Electronic Supplementary Material (ESI) for RSC Advances.
This journal is © The Royal Society of Chemistry 2019

Electronic supplementary information

‘Clean’ hydrolase reactions using commercial washing powder

Jie Zhang,[a] Fabio Tonin,[b] Wuyuan Zhang,[b] Peter-Leon Hagedoorn,[b] Lloyd Mallée[b] and Frank Hollmann*[b]

[a]Chongqing Key Laboratory of Catalysis & Functional Organic Molecules, College of Environment and Resources, Chongqing Technology and Business University, Chongqing 400067, China.

[b]Department of Biotechnology, Delft University of Technology, Van der Maasweg 9, 2629HZ Delft, The Netherlands. E-mail: F.Hollmann@tudelft.nl

Experimental procedures

Materials

Enzymatic laundry powder (Ariel, Compact Original, Barcode: 4084500833708) was made by Procter & Gamble International Operations SA and purchased from the local store. The inactivated ELP was obtained at 150 °C for 30 min. Unless indicated otherwise all chemicals were supplied by Sigma-Aldrich, Fluka, Acros or Alfa-Aesar in the highest purity available and used without further treatment.

Transesterification and esterification reactions

Unless mentioned otherwise, the reactions with ELP using ethyl acetate as solvent were performed in a reaction scale of 10 mL with a transparent Falkon (15 mL) at 60 °C under a rotating machine (neoLab Rotator mit Vortexer, C1 mode, 99 rpm), as shown in Figure S1. And the reactions with ELP using toluene as solvent were performed in a reaction scale of 10 mL with a transparent glass bottle (20 mL) at 60 °C under a magnetic stirrer (500 rpm). Aliquots were extracted by a pipette and analyzed by gas chromatography.

During the recyclability experiments, the Falkon was removed into a centrifuge (5810R, Eppendorf) and centrifuged at 10 °C, 5000 rpm for 10 min after each catalytic reaction. After centrifugation the supernatant was discarded. Continuously, fresh isopentyl alcohol solution was added in the Falkon containing ELP. The catalytic experiments were carried out in duplicates from two distinct samples unless indicated otherwise.

Hydrolysis reaction

The reactions were performed in a reaction scale of 2 mL with a transparent glass bottle (4 mL) at 60 °C under a magnetic stirrer (500 rpm). Aliquots were extracted by a pipette and analyzed by gas chromatography.

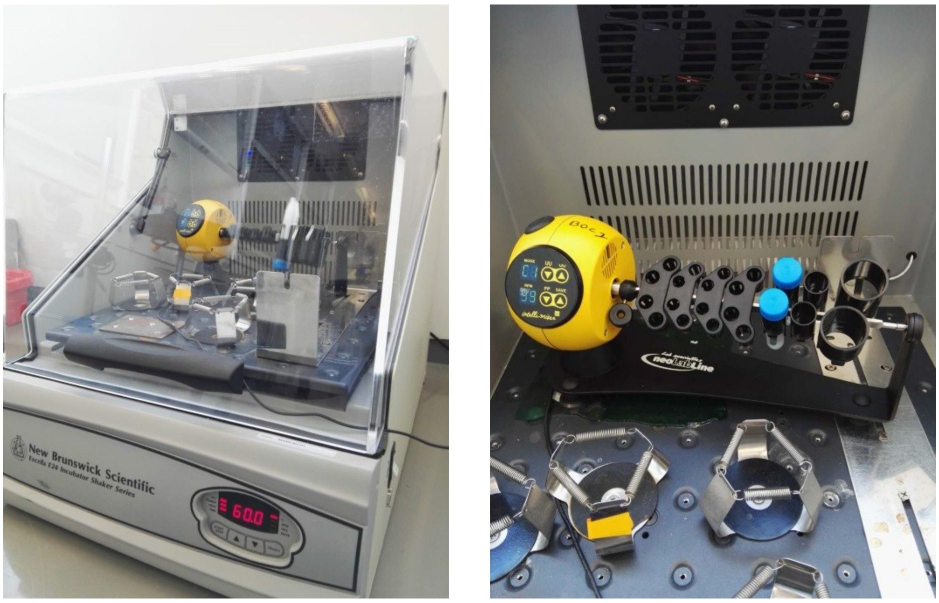

Figure S1. Picture of the reaction setup for rotating mode. Left: Whole reaction setup including the rotating machine. Right: Close-up of the rotating machine.

Kinetic resolution of 1-phenylethanol

The reactions with ELP were performed in a reaction scale of 10 mL with a transparent glass bottle (20 mL) at 60 °C under a magnetic stirrer (500 rpm). Reaction conditions: *c*(1-phenylethanol)=10 mM, *c*(vinyl acetate)=20 mM, toluene as solvent, 1000 mg ELP 48 h. Aliquots were extracted by a pipette and analyzed by gas chromatography. GC conditions: CP Chirasil Dex CB column (25m × 0.32mm × 0.25µm), FID detector, H_2 is the carrier gas. Temperature profile: 120 °C hold 2.6 min, 15 °C/min to 135 °C hold 3.3 min, 25 °C/min to 210 °C hold 0.5 min.

Epoxidation of styrene

The reactions with ELP using toluene as solvent were performed in a reaction scale of 10 mL with a transparent glass bottle (20 mL), *c*(styrene)=500 mM at 60 °C under a magnetic stirrer (500 rpm). Aliquots were extracted by a pipette and analyzed by gas chromatography. GC conditions: CP Wax 52CB column (25m × 0.25mm × 1.2µm), FID, N_2 is the carrier gas. Temperature profile: 110 °C hold 3 min, 20 °C/min to 150 °C hold 1 min, 20 °C/min to 200 °C hold 2.2 min, 30 °C/min to 250

°C hold 3 min. RT: Styrene, 3.566 min, 1-Octanol, 8.959 min, Styrene oxide, 10.354 min.

Semi-preparative scale production of isopentyl acetate

The reactions with ELP using ethyl acetate as solvent were performed in a reaction scale of 1 L, *c*(isopentyl alcohol)=500 mM and 150 g ELP. The reaction mixture was stirred using a KPG® stirrer (IKA Labortechnik) equipped with a Teflon stirrer (400 rpm) and the temperature was kept at 60 °C using an oil bath, as shown in Figure S2. The reaction was continued for 288 h.

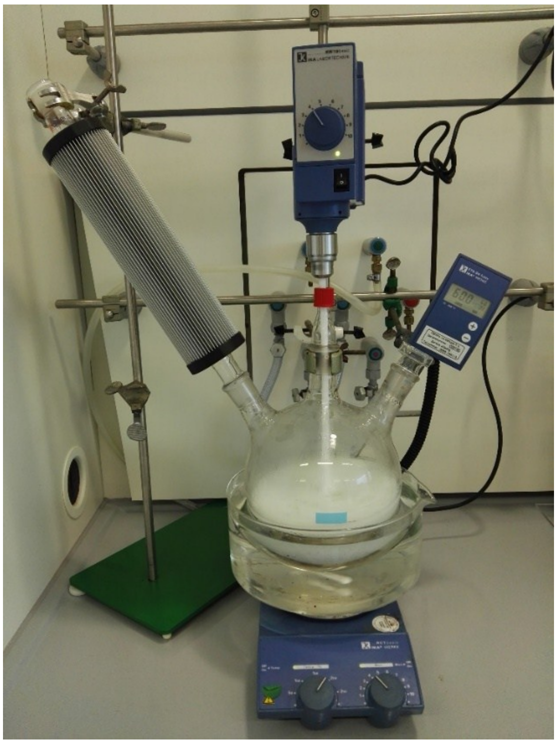

Figure S2. Picture of the reaction setup for semi-preparative scale production of isopentyl acetate.

The reaction mixture was filtered applying vacuum. The filtration residue was washed two times with ethyl acetate (2×50 mL). The filtered fluid was easily removed by evaporation under vacuum. After centrifugation (10 °C, 10000 rpm, 10 min), the raw product (31 mL) was obtained. For further purification, petroleum ether was added to this raw product to a volume of 45 mL. The pure chromatography systems (Reveleris® X2, Buchi) with a HP silica column (HP silica 20 µm, 24 g) were utilized to further purify isopentyl acetate. Petroleum ether was used as the mobile phase. The

flow rate was 25 mL/min. The sample solution was injected into the system by a syringe, and ELSD detector was used to monitor isopentyl acetate. According to the chromatogram the peak fraction of effluent of isopentyl acetate was collected automatically. Finally, isopentyl acetate containing fraction was concentrated under vacuum at 40 °C. The product was yielded in 1.02 g. An appropriate amount of product was dissolved in 600μL $CDCl_3$ and the ^1H NMR spectra were recorded on an Agilent 400 MHz NMR unit.

Quantification of products

Aliquots were withdrawn, added with ethyl acetate (containing 5mM of 1-octanol as internal reference), dried over $MgSO_4$ and analyzed by gas chromatography. Unless mentioned otherwise, all compounds in the reactions were analyzed by GC (model: SHIMADZU GC-2014 or GC-2010). Details of gas chromatograph and temperature profiles are shown in Table S1. Cinnamyl acetate and heptyl 2-phenylacetate were quantified by ^1H NMR, in which the crude mixture was dissolved in DMSO-D6 containing 5mM maleic acid as internal reference.

Table S1. Details of gas chromatograph and temperature profiles.

Product	Analysis column [a]	TR/min [b]	Temperature profile
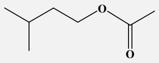 **Isopentyl acetate**	Column A	Isopentyl acetate, 6.469<br>Isopentyl alcohol, 7.505<br>1-Octanol, 11.786	75°C hold 3min, 15°C/min to 180°C hold 1min, 25°C/min to 230°C hold 1min.
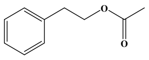 **2-Phenethyl acetate**	Column B	1-Octanol, 2.945<br>2-Phenylethanol,3.471<br>2-Phenethyl acetate, 4.623	110°C hold 2min, 20°C/min to 320°C hold 2min.
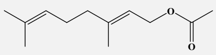 **Geranyl acetate**	Column B	1-Octanol, 2.945<br>Geraniol, 4.561<br>Geranyl acetate, 5.767	110°C hold 2min, 20°C/min to 160°C hold 2min, 20°C/min to 320°C hold 1min.
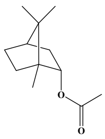 **Bornyl acetate**	Column B	1-Octanol, 2.945<br>Borneol, 3.945<br>Bornyl acetate, 4.916	110°C hold 2min, 20°C/min to 160°C hold 2min, 20°C/min to 320°C hold 1min.
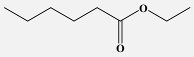 **Ethyl hexanoate**	Column A	Ethyl hexanoate, 7.197<br>1-Octanol, 10.955<br>Hexanoic acid, 14.411	75°C hold 2min, 15°C/min to 180°C hold 2min, 20°C/min to 230°C hold 6min.
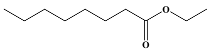 **Ethyl octanoate**	Column A	Ethyl octanoate, 9.53<br>1-Octanol, 10.955<br>Octanoic acid, 17.161	75°C hold 2min, 15°C/min to 180°C hold 2min, 20°C/min to 230°C hold 6min.
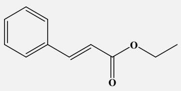 **Ethyl cinnamate**	Column B	1-Octanol, 2.945<br>trans-3-Phenylacrylic acid, 6.703<br>Ethyl cinnamate, 6.971	110°C hold 2min, 20°C/min to 160°C hold 2min, 20°C/min to 320°C hold 1min.
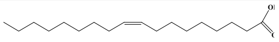 **Oleic acid**	Column B	1-Octanol, 3.204<br>Oleic acid, 12.913	110°C hold 3.4min, 25°C/min to 190°C hold 2.1min, 25°C/min to 230°C hold 4.1min, 30°C/min to 320°C hold 2min.

[a] Column A: CP Wax 52CB column (25m × 0.25mm × 1.2μm), FID, N_2 is the carrier gas; Column B: CP Sil 5CB column (50m × 0.53mm × 1.0μm), FID, N_2 is the carrier gas. [b] 1-Octanol (5mM in ethyl acetate) is used as internal standard except otherwise note.

Supplementary figures

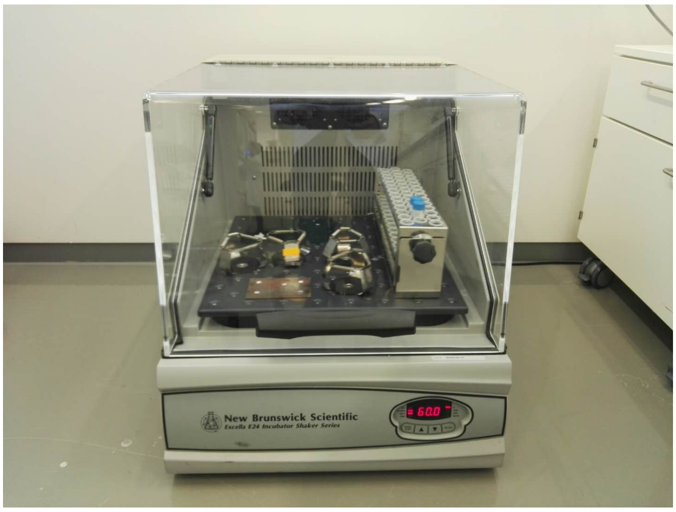

Figure S3. Picture of the reaction setup for shaking mode.

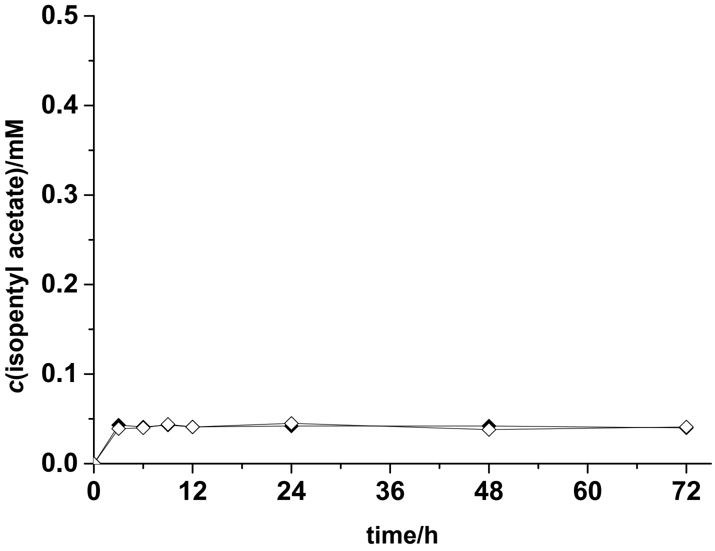

Figure S4. Time course of the isopentyl acetate formation with inactivated ELP (◆) and without ELP (◇). Conditions: 10 mL reaction scale, *c*(isopentyl alcohol)=1000 mM, ethyl acetate as solvent, 1000 mg ELP, 60 °C, shaking speed 180 rpm.

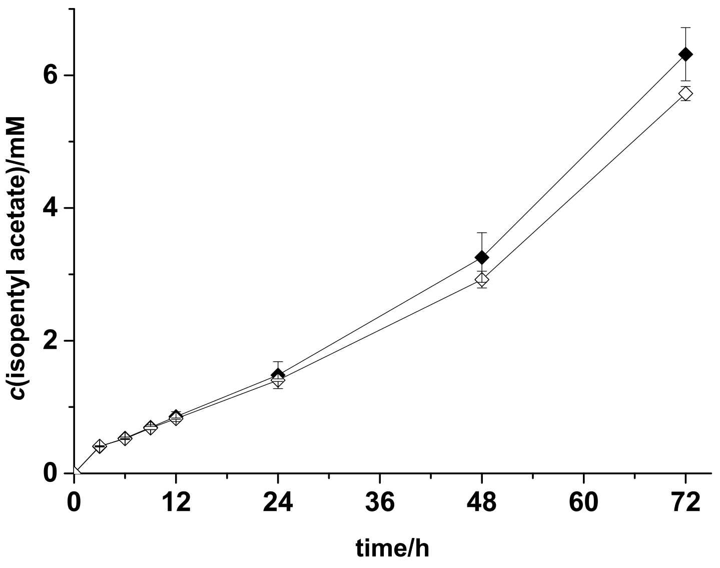

Figure S5. Time course of the isopentyl acetate formation with ELP (◆) and ground ELP (◇). Conditions: 10 mL reaction scale, *c*(isopentyl alcohol)=500 mM, ethyl acetate as solvent, 1000 mg ELP, 60 °C, shaking speed 180 rpm.

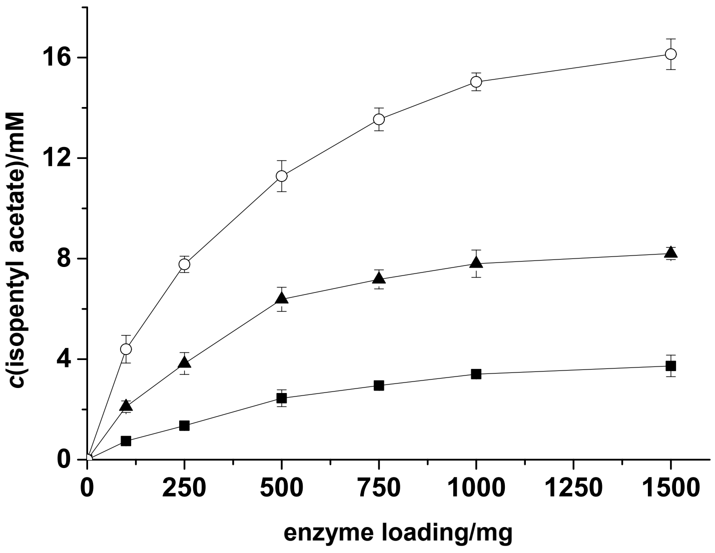

Figure S6. Effect of ELP loading on the transesterification of ethyl acetate. Time of 24 h (■), 48 h (▲), 72 h (○). Conditions: 10 mL reaction scale, *c*(isopentyl alcohol)=500 mM, ethyl acetate as solvent, 60 °C, rotating speed 99 rpm.

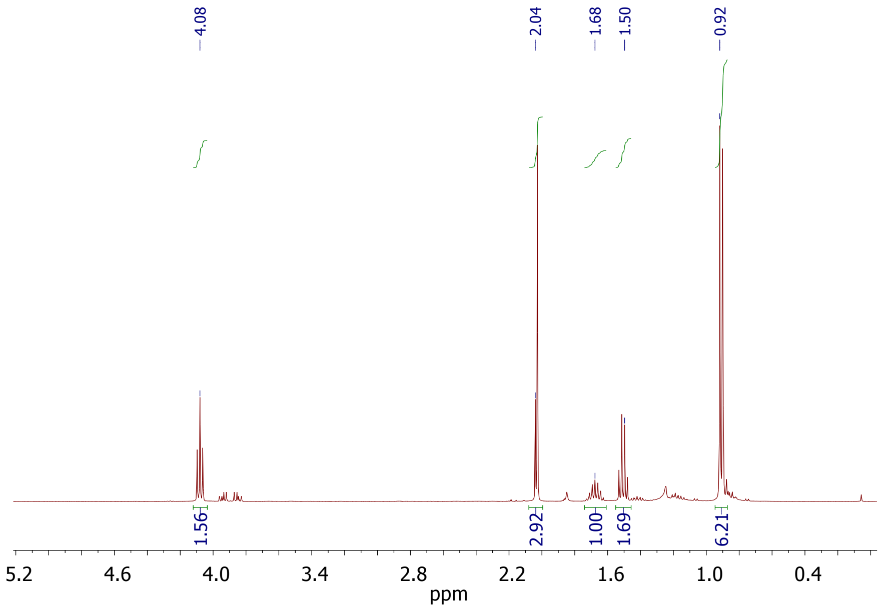

Figure S7. ^{1}H NMR (in $CDCl_3$) of isopentyl acetate from the semi-preparative scale synthesis after 288 h.

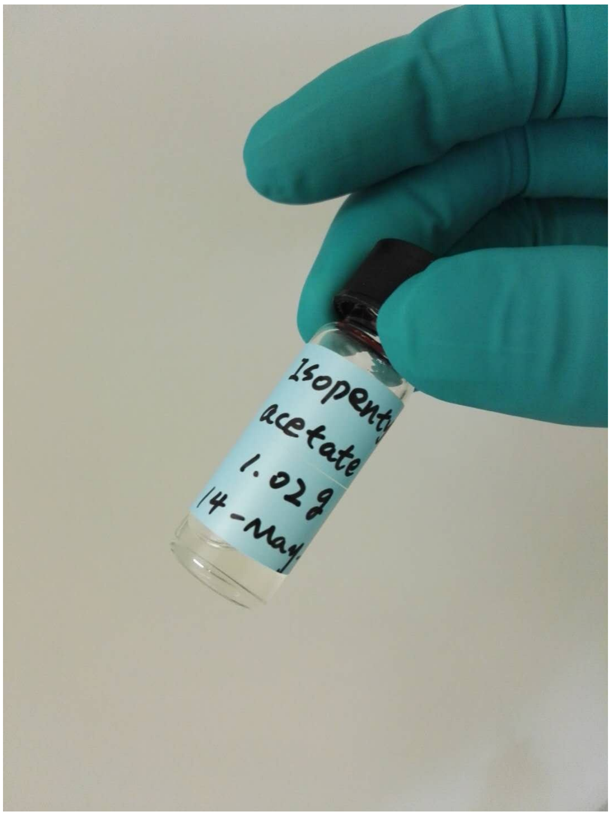

Figure S8. Image of isolated isopentyl acetate from the semi-preparative scale synthesis.

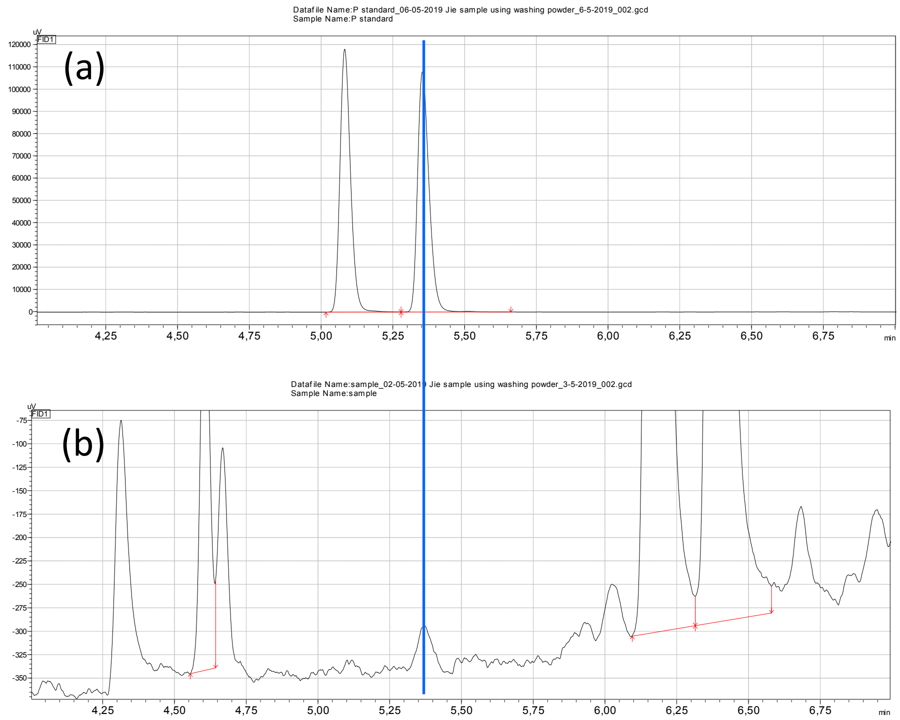

Figure S9. Representative GC chromatogram of commercial 1-phenylethyl acetate (a) and crude sample (b) from the reaction of kinetic resolution of 1-phenyl ethanol catalysed by ELP. Sample was taken after 48h.
